# Emergence of a New Delhi metallo-β-lactamase-1-producing *Pseudomonas aeruginosa* in Singapore

**DOI:** 10.1038/emi.2015.72

**Published:** 2015-11-25

**Authors:** Jeanette WP Teo, My-Van La, Roland Jureen, Raymond TP Lin

**Affiliations:** 1 Department of Laboratory Medicine, Division of Microbiology, National University Hospital, Singapore 119074, Republic of Singapore; 2 National Public Health Laboratory, Ministry of Health, Synapse, Singapore 138623, Republic of Singapore

## 

**Dear Editor,**

Among the clinically significant carbapenemases, the New Delhi metallo-β-lactamase (NDM) is one of the most formidable. NDM efficiently hydrolyses β-lactams and last-resort carbapenems. Hence, therapeutic options for infections by NDM-producers are restricted to a handful of antibiotics, such as colistin, tigecycline, and fosfomycin.^1^ NDM is predominantly associated with Enterobacteriaceae. This carbapenemase has also been described in *Acinetobacter* spp. but has been much less frequently detected in *Pseudomonas aeruginosa.*^1^

The first report of NDM-1 production in *P. aeruginosa* came from Serbia in 2011.^2^ It is now acknowledged that the NDM-1 gene is endemic to the Balkan states.^3,4^ NDM-producing *P. aeruginosa* has since been isolated from other European countries,^4,5,6,7^ as well as in India^8^ and Egypt.^9^ Local hospital laboratory surveillance suggests that approximately 12% of *P. aeruginosa* isolates are not susceptible to carbapenems.^10^ Previously, only producers of the metallo-β-lactamases (MBLs) VIM and IMP had been sporadically detected.^11^ Here, we describe the first observed case of NDM-1-producing *P. aeruginosa* in Southeast Asia.

*P. aeruginosa* was cultured from the endotracheal aspirate of a 90-year-old female patient with colon cancer in March 2015. The isolate exhibited multidrug resistance to carbapenems (meropenem, imipenem, minimum inhibitory concentrations (MICs) > 32 mg/L), cephalosporin (ceftazidime, cefepime, MICs > 256 mg/L), aminoglycosides (gentamicin, amikacin, MICs > 256 mg/L), and fluoroquinolones (ciprofloxacin, levofloxacin, MICs > 32 mg/L). The isolate was partially resistant to aztreonam (MIC 16 mg/L). Colistin susceptibility was observed at an MIC of 1 mg/L. Phenotypic testing for carbapenemases using the KPC/MBL Confirm Kit (Rosco Diagnostica A/S, Taastrup, Denmark) indicated the presence of an MBL.

Comprehensive polymerase chain reaction (PCR) screening for β-lactamase genes was performed.^12^ The isolate was positive for *bla*_NDM_ and determined to be *bla*_NDM-1_ by full-length gene sequencing. The isolate was negative for other MBLs (IMP, VIM, SPM, DIM, AIM) and for genes encoding class A carbapenemases (KPC, GES). TEM and CTX-M extended spectrum β-lactamases (ESBLs) were found to be present.

Plasmid analysis using S1 nuclease pulsed-field gel electrophoresis^13^ and spin column plasmid extractions (QIAprep Spin Miniprep Kit, QIAGEN, Valencia, CA, USA) did not the reveal the presence of plasmids. Southern blot analysis with a *bla*_NDM-1_ probe of S1 nuclease-treated DNA agarose plugs indicated that the probe hybridized to high molecular weight chromosomal DNA, suggesting that *bla*_NDM-1_ was situated on the chromosome (data not shown). Furthermore, solid media conjugation assays were performed to assess the transferability of *bla*_NDM-1_ from the clinical isolate to the azide-resistant recipient *Escherichia coli* J53. No transconjugants were obtained, suggesting the non-transmissibility of *bla*_NDM-1_ (at least to *E. coli*), again suggesting a chromosomal position. Because the isolate was highly resistant to most antibiotics, including rifampicin, this excluded the use of the rifampicin-resistant laboratory strain of *P. aeruginosa* for conjugation. Hence, we were unable to assess the intra-species transmissibility of *bla*_NDM-1_. *P. aeruginosa bla*_NDM-1_ may be present chromosomally^2,5,6^ or on a plasmid; in the latter context, *bla*_NDM-1_ is transmissible.^8^

The nucleotide sequences immediately flanking *bla*_NDM-1_ were determined by an inverse PCR and primer walking approach. Two copies of *bla*_NDM-1_ were detected and separated by an Insertion Sequence Common Region (IS*CR*) element (Figure 1). Sequencing of this IS*CR* element revealed a 97% nucleotide homology to IS*CR24*. IS*CR24* has been identified in the genetic environment of a novel *bla*_PME-1_ (*Pseudomonas aeruginosa* ESBL 1) and implicated in the acquisition of the ESBL by *P. aeruginosa*.^15^ This is not surprising because IS*CR* elements mediate the mobilization of almost every class of antibiotic resistance genes, including those encoding ESBLs and carbapenemases.^16^ IS*CR* elements, such as IS*CR1*, have been found to be associated with NDM-1 from *P. aeruginosa*.^3,5,7^ Jovcic *et al*.^3^ reported two copies of *bla*_NDM-1_ in *P. aeruginosa* (Figure 1), where it is presumed that the IS*CR1* element, as part of its rolling-circling mechanism of transposition, duplicates adjacent genetic segments.^16^ Because IS*CR* elements are known to construct extended clusters of antibiotic resistance genes on plasmids as well as on chromosomes,^16^ it would be interesting to investigate the presence of other resistance determinants surrounding *bla*_NDM-1_ and the IS*CR24*-like region that may contribute to its multidrug resistance.

After this isolate was identified, two other NDM-1-producing *P. aeruginosa* isolates with antibiograms identical to the initial isolate were cultured from the sputum samples of two other patients in April and May 2015. One of the isolates was from a 58-year-old male with intracranial bleeding from a ruptured aneurysm, and the other was from an 84-year-old female with a femur fracture, complicated by pancreatitis and small intestine perforation. The genetic relatedness of the three NDM-1 *P. aeruginosa* isolates was investigated by DiversiLab rep-PCR fingerprinting (bioMérieux, Marcy l'Etoile, France), which revealed that the rep-PCR profiles were indistinguishable, suggesting that the three isolates were clonal in nature. PCR mapping and sequencing of the two latter isolates reveal a *bla*_NDM_ genetic context identical to that of the first isolate. Because all three patients stayed in the same surgical intensive care unit, the detection of indistinguishable NDM-1-positive *P. aeruginosa* suggested a transmission event.

In summary, this is the first report of the emergence of NDM-1 in *P. aeruginosa* in Southeast Asia in an unusual genetic context. The apparent intra-ward transmission of this extremely drug-resistant isolate highlights the gravity of this escalating public health issue.

### Nucleotide sequence accession number

The *bla*_NDM-1_ sequence from the initial *P. aeruginosa* isolate has been deposited into Genbank under the accession number KT364224.

## Figures and Tables

**Figure 1:**
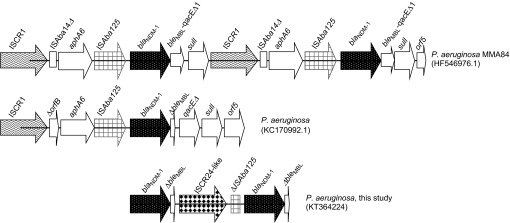
Comparative schematic diagrams of the diverse genetic organizations of *bla*_NDM-1_ in *P. aeruginosa*. The selected *bla*_NDM-1_ sequences are chromosomally located and associated with IS*CR* elements and class I integrons.^3,5^ Typical common genetic features surrounding *bla*_NDM-1_ are the insertion sequence IS*Aba125* and the bleomycin resistance gene (*ble*_MBL_).^14^
